# Upregulating β-hexosaminidase activity in rodents prevents α-synuclein lipid associations and protects dopaminergic neurons from α-synuclein-mediated neurotoxicity

**DOI:** 10.1186/s40478-020-01004-6

**Published:** 2020-08-06

**Authors:** Oeystein R. Brekk, Joanna A. Korecka, Cecile C. Crapart, Mylene Huebecker, Zachary K. MacBain, Sara Ann Rosenthal, Miguel Sena-Esteves, David A. Priestman, Frances M. Platt, Ole Isacson, Penelope J. Hallett

**Affiliations:** 1 Neuroregeneration Institute, McLean Hospital / Harvard Medical School, Belmont, MA USA; 2 Current address: Department of Neurology, Brigham and Women’s Hospital, Boston, MA USA; 3 Department of Pharmacology, University of Oxford, Oxford, UK; 4 Current address: Institute of Innate Immunity, Medical Faculty, University of Bonn, Bonn, Germany; 5 Department of Neurology, University of Massachusetts Medical School, Worcester, MA USA

**Keywords:** α-Synuclein, β-Hexosaminidase, Sandhoff disease, Parkinson’s disease, Neuroprotection, Lipid binding

## Abstract

Sandhoff disease (SD) is a lysosomal storage disease, caused by loss of β-hexosaminidase (HEX) activity resulting in the accumulation of ganglioside GM2. There are shared features between SD and Parkinson’s disease (PD). α-synuclein (aSYN) inclusions, the diagnostic hallmark sign of PD, are frequently found in the brain in SD patients and HEX knockout mice, and HEX activity is reduced in the substantia nigra in PD. In this study, we biochemically demonstrate that HEX deficiency in mice causes formation of high-molecular weight (HMW) aSYN and ubiquitin in the brain. As expected from HEX enzymatic function requirements, overexpression in vivo of HEXA and B combined, but not either of the subunits expressed alone, increased HEX activity as evidenced by histochemical assays. Biochemically, such HEX gene expression resulted in increased conversion of GM2 to its breakdown product GM3. In a neurodegenerative model of overexpression of aSYN in rats, increasing HEX activity by AAV6 gene transfer in the substantia nigra reduced aSYN embedding in lipid compartments and rescued dopaminergic neurons from degeneration. Overall, these data are consistent with a paradigm shift where lipid abnormalities are central to or preceding protein changes typically associated with PD.

## Introduction

Age-related neurodegenerative diseases, such as Parkinson’s disease (PD), have many etiological roots that converge on mitochondrial, lipid, protein and inflammatory pathological mechanisms [16, 19]. Lysosomal storage diseases (LSDs) are a class of more than 70 metabolic disorders typically characterized by autosomal recessive loss-of-function mutations in lipolytic hydrolases and the concurrent accumulation of the associated lipid substrate [32, 34]. Among these enzymes is β-hexosaminidase (HEX), which is responsible for turnover of the glycosphingolipid ganglioside GM2 [51]. There are three separate isoforms of HEX, generated by combined expression from the two gene loci *HEXA* and *HEXB*: the HEX A isoform, consisting of a HEXα +HEXβ heterodimer, and two homodimers consisting of subunits HEXβ+HEXβ (HEX B) and HEXα+HEXα (HEX S) [2]. Impaired GM2 catabolism causes two clinically similar diseases, which are classified depending on which locus is mutated: Tay-Sachs disease (*HEXA*) and Sandhoff disease (SD) (*HEXB*) [6]. In both cases, the net effect is loss of the HEX A + B heterodimer, and both diseases are characterized by neuronal swelling and degeneration caused by elevated ganglioside storage [41].

PD, which affects nearly 4 in 100 people over the course of a lifetime [26], characterized by progressive loss of dopaminergic neurons of the substantia nigra pars compacta (SNpc) and concurrent aggregation of the synapse-associated protein α-synuclein (aSYN) [35], shares many pathophysiological features associated with LSDs. *GBA1* encodes the lysosomal hydrolase glucocerebrosidase (GCase), responsible for catabolizing the glycosphingolipids glucosylceramide and glucosylsphingosine, and it is the causative gene in the LSD Gaucher disease (GD) [14]. Haploinsufficiency caused by carrier mutations in *GBA1* constitute the strongest genetic risk factor for PD, accounting for 7–10% of cases [46], and exome-wide analysis of 53 LSD-related genes, including *HEXB,* showed an overall association of rare variants with PD - although the variation was also high in the healthy subject population [37]. Genome-wide association studies have also identified PD risk variants in three additional lysosomal genes (*GUSB*, *GRN,* and *NEU1*) [56], and cell-type specific enrichment of lysosomal genes have demonstrated associations to PD heritability when selecting for astrocytic, microglial and oligodendroglial subtypes [36]. Enzymatic activity of both GCase and HEX are progressively lost with age in PD [17, 38], with HEX-activity only showing significant downregulation compared to healthy subject controls in patients aged 80 years or older [17, 28]. GD- [52] and SD [48] patient brains show evidence of aSYN aggregation. Experimentally, both pharmacological inhibition of GCase and targeted deletion of the *HEXB* locus produce aSYN aggregation in mice [9, 39, 49]. In human PD brains and mouse models characterized by impaired glycolipid metabolism, aSYN exists within lipid compartments and forms high molecular weight (HMW) species undetectable to conventional biochemical assays [7, 20, 45]. Furthermore, the presence of aSYN pathology in GD- and SD- patient samples and experimental models reducing either GCase or HEX activity is interesting because it highlights the role of lysosomal enzymes in this classic pathology. The overall importance of lipid catabolism and turnover is highlighted by the recent findings that the major components of the pathology for PD, Lewy bodies, are lipids. Such Lewy bodies, coated by aSYN, were shown to be predominantly composed of a wide variety of lipid-containing subcellular components, including vesicles, organelles, and disordered membranes [44].

Gangliosides are enriched in neurons, where they predominantly localize to the outer leaflet of the plasma membrane in specialized lipid microdomains termed ‘lipid rafts’ [47], and the primary proposed physiological interaction of aSYN with gangliosides is localization to synaptic terminals through binding of lipid raft domains [12]. Though originally believed to be exclusively neuronal constituents, relative abundances of certain species of gangliosides (e.g. GM3) are higher in astrocytic lipid rafts than in neurons [1], and it is possible GM2/GM3 localization plays an additional role in neuron-glia interactions.

In this study, we first aimed to biochemically characterize aSYN changes in the transgenic *HEXB*^*−/−*^ SD mouse model with GM2 gangliosidoses. Given that complete loss of ability to hydrolyse GM2 in the lysosome because of *HEXB* loss causes PD-like accumulation of aSYN [9, 49], we hypothesized that increased HEX activity could protect neurons from neurotoxicity associated with experimentally induced aSYN accumulation. To this end, we utilized a recombinant adeno-associated viral (AAV6) vector strategy to specifically overexpress aSYN ~ 3-fold in the vulnerable dopaminergic neurons of the rat SNpc, which causes progressive degeneration of the nigrostriatal circuit with concurrent aSYN accumulation [21, 50, 54], combined with AAV6-mediated expression of *HEXA* and *HEXB*. We found that upregulated HEX activity rescued degeneration of rat dopaminergic neurons and limited interactions between lipid compartments and overexpressed aSYN.

## Methods

### Recombinant adeno-associated viral vector preparation

The AAV6-aSYN vector contains a coding sequence for human WT-aSYN under the control of a synapsin-1 promoter. The titer of AAV6-aSYN stock was 2.42 × 10^12^ viral genome copies (vgcs) / mL. AAV6 promoter-free empty vector (EV) control virions were acquired from Virovek (Hayward CA, USA), and the final titer was estimated to be 2 × 10^12^ by qPCR, followed by SDS-PAGE electrophoresis. AAV6-HEXA and AAV6-HEXB vectors contained coding sequences for human HEX A and HEX B under the control of a CBA promoter region. The final titer for AAV6-HEXA was 9.32 × 10^11^ genome copies / mL, and for AAV6-HEXB it was 8.7 × 10^11^ genome copies / mL. The AAV6-GFP construct utilized as control in the GM3 HPLC-measurements was under control of a CBA promoter region, with a final titer of 1.06 × 10^12^. The titers were estimated by real-time quantitive PCR using primers and a Taqman probe to the bovine growth hormone poly-adenylation site present in both HEXA and HEXB vectors.

### Animals

All animal procedures were performed in accordance with the guidelines of the National Institute of Health and were approved by the Institutional Animal Care and Use Committee (IACUC) at McLean Hospital, Harvard Medical School. Animals were housed per standard conditions, in a dark/light cycle of 12 h, with ad libitum access to food and water. 12-week-old male (*N* = 4) and female (*N =* 4) WT mice (C57BL/6, Jackson Laboratories, and 11–14-week-old male and female *HEXB*^*−/−*^ Sandhoff mice [42] (*N* = 8) (obtained from Dr. Miguel Sena-Esteves) were used in this study. 235-260 g naïve Sprague-Dawley rats (*N* = 30) (Charles River Laboratories) were used for all stereotactic surgical experiments.

### Stereotactic delivery of AAV6 to the rat substantia nigra pars compacta

Female Sprague-Dawley rats weighing 235-260 g were used for all stereotactic surgical experiments. These experiments had previously been approved by the McLean Hospital Institutional Animal Care and Use Committee (IACUC). Stereotaxic coordinates for the surgeries were adapted from prior studies [10]. Animals received a pre-anesthetic mix of 0.08mg/kg Atropine and 1.7mg/kg Acepromazine delivered intramuscularly 15 min prior to anesthesia with xylazine and ketamine (5.4 mg/kg and 72 mg/kg, respectively). Prior to fixation in the stereotactic frame, each animal received 1 mg/kg Metacam delivered subcutaneously. During the surgery, animals received oxygen at 0.15 mL/min. The animals were placed in a stereotaxic frame (Kopf), where a 10 μl Hamilton syringe and 31 gauge needles were used as a delivery system. All injections were made into the substantia nigra pars compacta using the following anteroposterior (AP), mediolateral (ML), and dorsoventral (DV) coordinates as previously verified [23, 54]: AP − 5.2 mm, ML − 2.0 mm, DV − 7.2 mm. The injection titers of the viruses were 2 × 10^12^ vgc/mL for aSYN, and 8.7 × 10^11^ vgc/mL for HEX A, B, and EV, and 3 μl were injected per site. For the GM3 measurements, AAV6-GFP was utilized as a control, with an injection titer of 8.70 × 10^11^ vgc/mL. The viral deliveries were done at 0.4 μl/min using a microinfusion pumps (Stoelting), with a 3 min wait, followed by 0.5 mm withdrawal of needle, followed by an additional 1 min wait, before the needle was slowly withdrawn completely. Anesthesia was reversed post-operatively by Antisedan delivered intramuscularly at 1 mg/kg.

### Immunohistochemistry

Animals were terminally anesthetized with an overdose of sodium pentobarbital (150 mg/kg, intraperitoneal delivery) and perfused intracardially with ice-cold heparinized saline (0.1% heparin in 0.9% saline) followed by paraformaldehyde (PFA) (4% in PBS). The brains were removed and postfixed for 24 h in 4% PFA. Following postfixation, the brains were equilibrated in 20% sucrose in PBS, sectioned at 40 μm on a freezing microtome, and serially collected in PBS. Sections were washed three times in PBS 0.1% Triton-X, blocked in 10% normal goat serum in PBS 0.3% Triton-X, and primary antibodies were applied overnight at 4C. Primary antibodies used were rabbit anti-tyrosine hydroxylase (Pel-Freez Biologicals Cat# P40101, RRID:AB_2313713), mouse anti-aSYN (human) (Millipore Cat# 36–008, RRID:AB_310817), mouse anti-aSYN (pan species) (BD Biosciences Cat# 610786, RRID:AB_398107), mouse anti-LAMP-1 (Santa Cruz Biotechnology Cat# sc-65,236, RRID:AB_831070), and mouse anti-HEX A (Santa Cruz Biotechnology Cat# sc-376,777). After primary antibodies, tissues were washed three times in PBS 0.1% Triton-X, and secondary antibodies were applied at room temperature in dark for 60 min. Secondary antibodies (goat anti-mouse and anti-rabbit) were from Thermo-Fisher Scientific (Alexa Fluor 488 (RRID:AB_26633275) & 568 (RRID:AB_141371). Coverslipping was done utilizing Mowiol (Sigma-Aldrich Cat# 81381).

### Histochemical hexosaminidase activity assay

Animals were decapitated following an overdose of sodium pentobarbital (150 mg/kg, intraperitoneal delivery), and the brains sectioned at 12 μm thickness through the coronal plane by cryostat. Following postfixation in 4% PFA for 10 min at room temperature, β-hexosaminidase activity was detected histologically in fresh-frozen, cryosectioned rat coronal sections using Naphthol AS-BI-N-acetyl-β-D-glucosaminide (Sigma Aldrich Cat# N4006) as previously described [8, 24]. Briefly, sections were incubated in 0.25 mM Naphthol AS-BI-N-acetyl-β-D-glucosaminide in 50 mM Sodium Phosphate Citrate Buffer (pH 5.2) with hexazotized pararosaniline for 6 h at RT, prior to washing and coverslipping.

### Stereological estimates of tyrosine hydroxylase^+^ dopaminergic neurons and densitometric analysis of the dorsolateral striatum

Number of tyrosine hydroxylase (TH)-positive dopaminergic neurons of the substantia nigra pars compacta was estimated by an unbiased stereological quantification method using the optical fractionator principle [5, 18] embedded in the Stereo Investigator v2019.1.3 software (MBF Bioscience), a motorized stage (LE2-MB, LUDL electronics) and a top-mounted camera (ORCA Flash4.0LT, Hamamatsu) with the light microscope (Axio Imager M2, Carl Zeiss Microscopy). Every sixth section throughout the whole rostro-caudal axis of the substantia nigra was included, region of interest was outlined with a 2.5x objective and counting was performed using a 40x objective. Random start and systematic sampling were applied. Counting parameters were adjusted to achieve at least 100 counts per hemisphere. All quantifications and analysis were performed by a single blinded investigator. A coefficient of error (Gundersen) [15] of 0.1 was accepted. Data are expressed as % of the non-injected control hemisphere. Striatal TH^+^-density was quantified using 4–6 coronal sections per brain, covering the whole rostro-caudal axis of the striatum. Sections were tile-scanned with a 20x objective using a BZ-X700 fluorescent microscope (Keyence), and the dorsolateral aspect of the striatum was analyzed in ImageJ (NIH) in the indicated regions (see Fig. 3a), quantifying average TH^+^ pixel values. Data were then normalized as % of the non-injected control hemisphere in each section.

### Biochemistry and western blotting

WT- and SD mice were terminally anesthetized with an overdose of sodium pentobarbital (150 mg/kg, intraperitoneal delivery) and perfused intracardially with ice-cold heparinized saline (0.1% heparin in 0.9% saline). Brains were extracted and separated by the midline, and half-brains were spot dissected for substantia nigra and the dorsolateral aspect of the striatum prior to being frozen on dry ice for biochemical assays. Lysis of whole-brain tissues was carried out for 30 min on ice in lysis buffer (150 mM NaCl, 50 mM Tris pH 7.6, 1% Triton X, 2 mM EDTA) with protease/phosphatase inhibitors added (Halt Protease & Phosphatase Inhibitor Cocktail (100X), Thermo Fisher Scientific Cat# 1861284), and the lysates sonicated for 30 s (5 s pulses, on ice). Membrane-enriched fractions were pelleted in 1% Triton-X by ultracentrifugation (100,000xG, 1 h, 4C), and reconstituted in lysis buffer supplemented 2% SDS. The supernatant from this initial separation constituted the cytosol-enriched fractions. After 2% SDS-supplemented lysates were centrifuged again (100,000xG, 1 h, 4C), the supernatant was collected as the membrane-enriched fraction. Equal amounts of protein from each fraction was separated using polyacrylamide gel electrophoresis (4–20% Criterion Tris-HCl Protein Gel, Bio-Rad Cat# 3450032). Primary antibodies included mouse anti-aSYN (pan species) (BD Biosciences Cat# 610786, RRID:AB_398107), rabbit anti-phospho-aSYN (S129) (Cell Signaling Technology Cat# 23706, RRID:AB_2798868), rabbit anti-ubiquitin (Agilent Cat# Z0458, RRID:AB_2315524), and chicken anti-GAPDH (Millipore Cat# AB2302, RRID:AB_10615768). Protein content of each sample was determined using a bicinchoninic acid (BCA) assay kit (Thermo Scientific Cat#23225). For delipidation, lysates were heated on a heating block (65C) for 16 h prior to addition of Laemmli-buffer, as previously described [45]. Secondary, HRP-conjugated antibodies were anti-mouse (Trueblot Ultra) (Rockland Cat# 18–8817-33) anti-chicken (Affinipure) (Jackson Immunoresearch Laboratories Cat# 103–035-155), and anti-rabbit (Thermo Fisher Scientific, A-21206). Chemiluminescent development was done using Westernbright Sirius (Advansta Cat# K-12043-D20), and imaging was done on a Gel Doc XR+ system (Bio-Rad Cat# 1708195). To ensure linearity of signal, only images without saturated pixels were selected for quantifications using the densitometric western blot plug-in in ImageJ (NIH).

### Ganglioside GM3 analysis by NP-HPLC

Ganglioside GM3 content from dissected rat substantia nigra (SN) injected with AAV6-HEX+aSYN and AAV6-GFP + aSYN were analyzed as previously described [17, 29]. Briefly, glycosphingolipids (GSLs) were extracted from rat SN with chloroform/methanol overnight at 4 °C and further purified using solid-phase C18 columns (Telos, Kinesis, UK). GSLs were dried down under a stream of nitrogen at 42 °C and treated with recombinant ceramide glycanase (rEGCase, prepared by Genscript and provided by Orphazyme, Denmark) to obtain oligosaccharides from complex GSLs. Liberated glycans were then fluorescently labelled with anthranillic acid (2AA). Excess 2AA label was removed using DPA-6S SPE columns (Supelco, PA, USA). Purified 2AA-labelled glycans were separated and quantified by normal-phase high-performance liquid chromatography (NP-HPLC) as previously described [29]. The NP-HPLC system consisted of a Waters Alliance 2695 separations module and an in-line Waters 2475 multi λ-fluorescence detector set at Ex λ360nm and Em λ425nm. The solid phase used was a 4.6 × 250 mm TSK gel-Amide 80 column (Anachem, Luton, UK). A 2AA-labelled glucose homopolymer ladder (Ludger, UK) was used to determine the glucose unit values (GUs) for the HPLC peaks. GM3 was identified by its GU value and quantified by comparison of integrated peak areas with a known amount of 2AA-labelled BioQuant chitotriose standard (Ludger, UK). Results were normalized to protein content, determined using bicinchoninic acid (BCA) assay.

### Lysosomal β-hexosaminidase activity measurements in rat substantia nigra

Lysosomal β-hexosaminidase activities were assayed fluorometrically using an artificial sugar-substrate conjugated with the fluorophore 4-methylumbelliferone (4-MU). The substrate utilized to assess β-hexosaminidase activity was 3 mM 4-MU N-acetyl-β-D-glucosaminide in 200 mM sodium citrate buffer, pH 4.5, 0.1% TritonX-100. The digests (in triplicate) containing tissue homogenate in PBS with 0.1% TritonX-100 and artificial 4-MU substrate were incubated at 37 °C for 30 min. The reaction was stopped by adding cold 0.5 M Na_2_CO_3_ (pH 10.7). The released fluorescent 4-MU was measured in a FLUOstar OPTIMA plate reader (BMG Labtech, Ortenberg, Germany) with an excitation at 360 nm and emission at 460 nm. A standard curve of free 4-MU was used to calculate the enzyme activity. Results were normalized to protein content.

### Statistics

Graphpad Prism (v8) was used for all statistics (GraphPad Software, San Diego, California, USA). Two-tailed, parametric t-tests, or matched sample t-tests, were utilized for single comparisons as indicated in figure legends. In cases of significantly different variances, the Welch’s correction was applied. For correlation analysis, Pearson’s r was computed. N (total independent replicates per group) and *p* values are indicated in figure legends. All graphs include mean with individual data points shown. Outlier analysis was done using the ROUT method embedded in Prism with a Q = 1%.

## Results

### ß-hexosaminidase-deficient Sandhoff disease model mice exhibit robust alpha-synuclein and ubiquitin accumulation in the brain

**Fig. 1:**
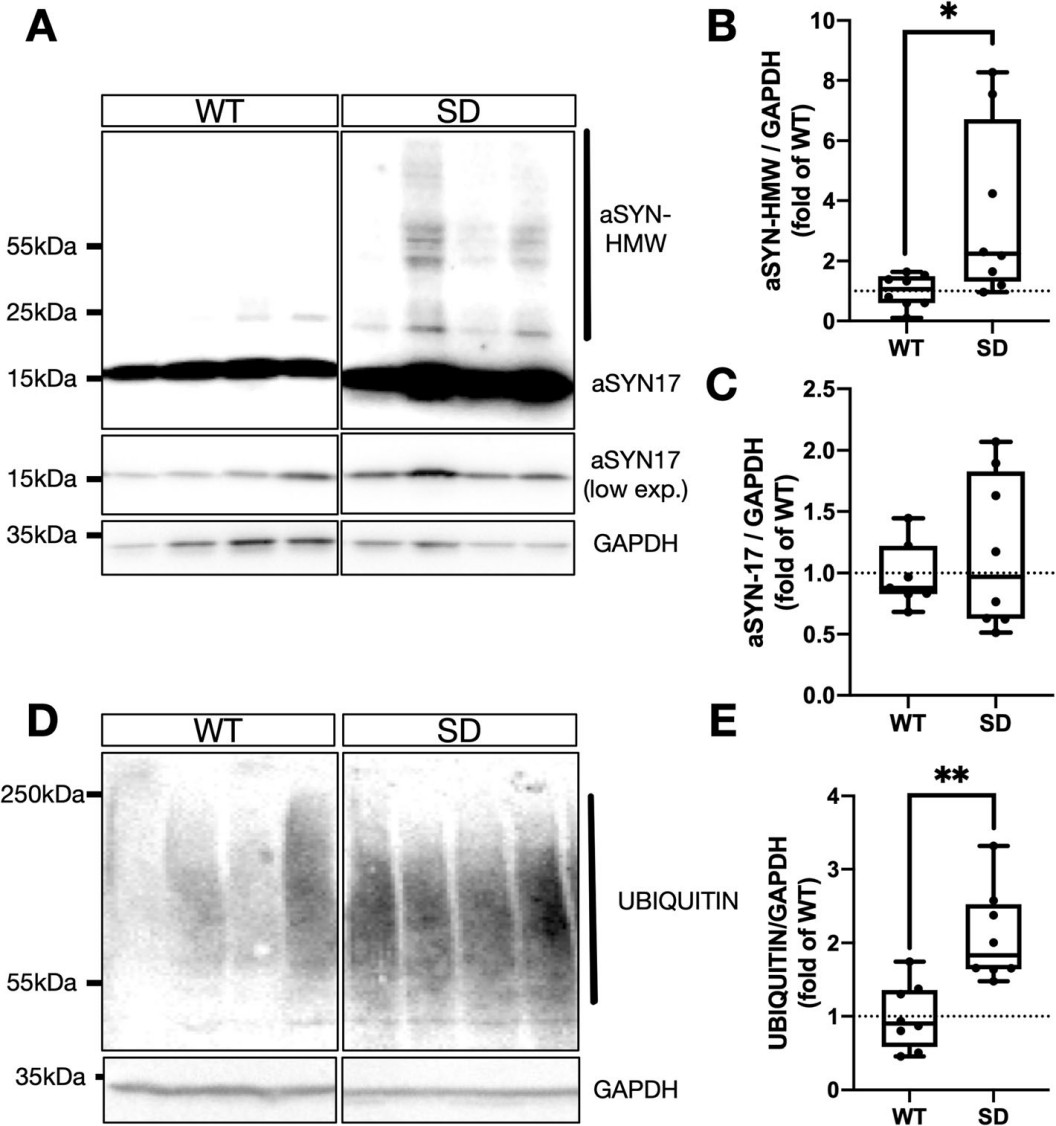
High molecular weight aSYN and ubiquitin accumulation accompany HEX-deficiencies in the Sandhoff mouse model. **a** Representative immunoblot of high molecular weight (HMW) aSYN in whole-brain homogenates from age-matched WT and SD mice. GAPDH is shown as loading control. **b** Densitometric quantifications of HMW-aSYN (>17kDA) of samples treated as in (**a**), normalized by GAPDH and expressed as folds of WT (*:*p* < 0.05, Welch’s t-test). **c** Densitometric quantifications of monomeric aSYN17 over GAPDH. **d** Representative immunoblot of ubiquitin in age-matched WT- and SD mouse whole-brain. GAPDH is shown as loading control. **e** Densitometric quantifications of total ubiquitin over GAPDH, expressed as folds of WT (***:*p* < 0.001, two-tailed t-test). Box plots show median, 95% CI and range, with individual data points shown for all graphs, *n* = 7–8 / group

To assess the impact of loss of lysosomal hydrolase ß-hexosaminidase (HEX) activity and concurrent ganglioside GM2 accumulation on alpha-synuclein (aSYN) homeostasis, total aSYN content was measured in whole brain samples collected from *HEXB*-deficient Sandhoff disease (SD) mice. Relative to age-matched WT littermates, SD whole-brains contained significantly elevated total (~ 2.5-fold) high molecular weight (HMW) aSYN species (Fig. 1a, b). Levels of monomeric ~ 17 kDa aSYN (aSYN17) were unchanged comparing the two cohorts (Fig. 1c). Prior histological studies have described ubiquitin inclusions in the SD mouse model [9, 49]. To provide biochemical evidence, WT- and SD whole-brain lysates were probed for ubiquitin (Fig. 1d). A significant ~ 2.5-fold increase in ubiquitin was detected in membrane-enriched SDS-soluble fractions (Fig. 1e). Ubiquitin accumulation was also verified by immunofluorescent co-labeling of aSYN and ubiquitin in SD mouse SNpc, which showed the presence of numerous aSYN+/ubiquitin+ inclusions (SFIG. 1A). Interestingly, the majority of larger ubiquitin+ inclusions did not contain aSYN+ immunoreactivity.

### Neuron-targeted overexpression of ß-hexosaminidase subunits A and B significantly increases lysosomal enzymatic activity and increases GM2 turnover

**Fig. 2:**
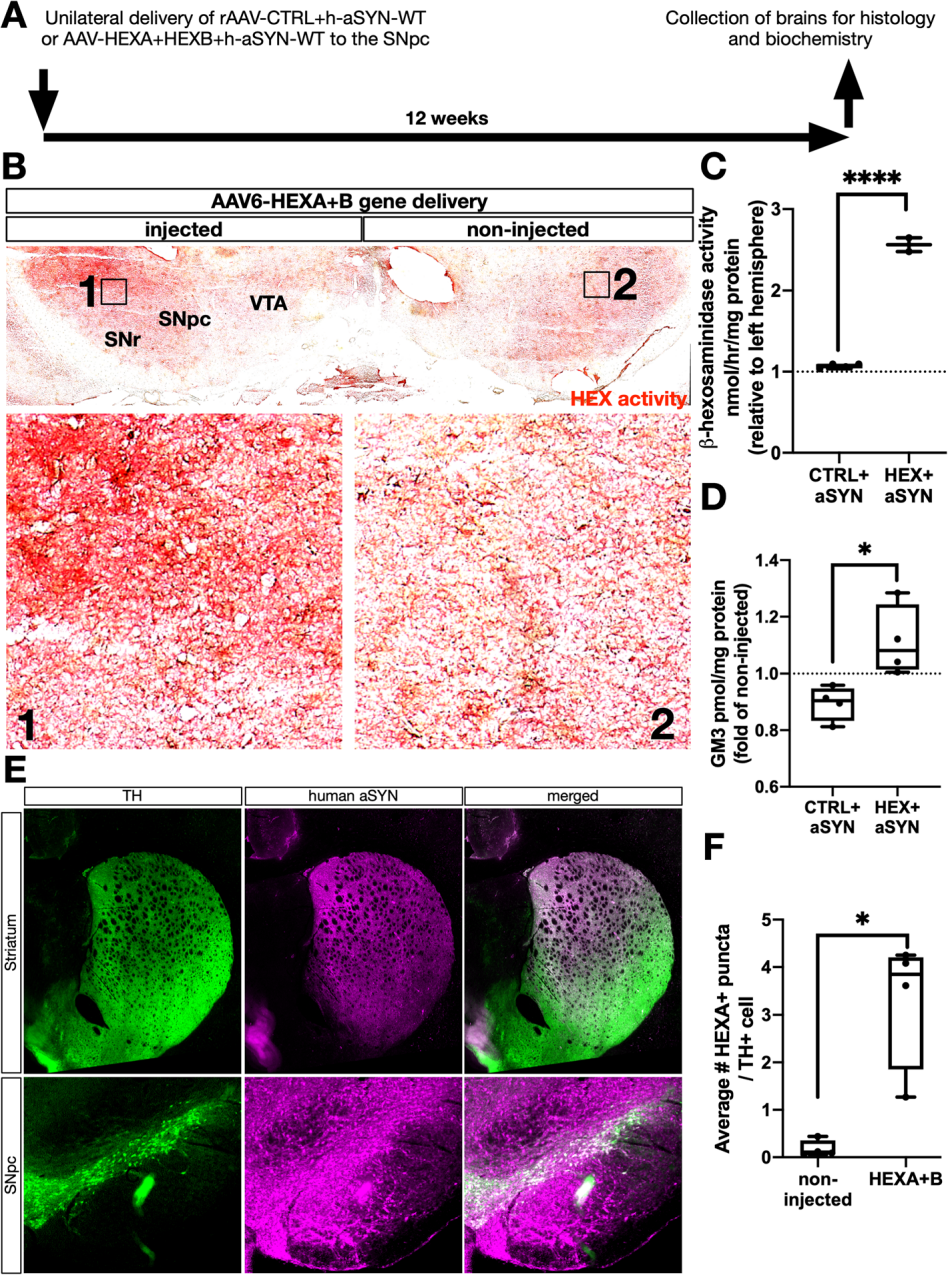
AAV6-expressed HEX subunits A and B localize to lysosomes and boost enzymatic activity in the rat substantia nigra. **a** Adult rats received unilateral SNpc injections of AAV6s expressing either aSYN and a control vector, or aSYN+HEX subunits A and B. Biochemical and histological measurements were collected at 12 weeks post-injection, and comparisons were made between injected- and non-injected control hemispheres for each animal. **b** HEX activity histochemical detection assay (red precipitate) in a rat injected with AAV6s expressing HEX subunits A and B. (SNr = substantia nigra pars reticulata, VTA = ventral tegmental area). Boxed inserts are shown in higher magnification below. **c** Lysosomal HEX activity measured by artificial 4-MU-substrate in dissected SN from rats injected with CTRL+aSYN (*n* = 4) and HEX+aSYN (*n* = 2) (****:*p* < 0.0001, two-tailed t-test). **d** HPLC measurements of GM3 in spot-dissected ventral midbrain from control+aSYN and HEX+aSYN injected rats, normalized by the non-injected hemispheres (*n =* 4 / group). **e** Representative immunofluorescent micrograph showing AAV6-overexpressed human WT aSYN (magenta) in dopaminergic neurons (TH+) (green) in the rat striatum and SNpc. **f** Quantifications of # HEX A+ puncta / TH+ cell in images as in SFIG. 3 (*n* = 4 / group) (*:*p* < 0.05, Welch’s t-test)

To determine whether an upregulation of HEX activity could protect nigrostriatal dopaminergic neurons from neuron-targeted AAV6-mediated human WT-aSYN toxic overexpression in the rat substantia nigra pars compacta (SNpc), which normally produces progressive loss of tyrosine hydroxylase (TH^+^) dopaminergic neurons [21, 54], we overexpressed HEX subunits A and B using AAV6 in the same region (Fig. 2a). HEX enzymatic function requires assembly of a HEXA+B heterodimer with subsequent translocation to the lysosomal lumen [2] (‘HEX’ refers to the combined expression of both AAV6-HEXA- and B vectors.) To localize differential enzymatic activity spatially in AAV6-HEX injected rat brain, a histochemical HEX activity stain was used as previously described [9] (Fig. 2b). In HEXA+B injected rat SNpc, there was a clear increase of HEX activity in the targeted region relative to the non-injected contralateral hemisphere. This was verified by enzymatic activity measurements of lysosomal HEX in dissected SN from a subset (*n* = 2) of HEX-injected rats relative to CTRL+aSYN injected animals (*n* = 4) (Fig. 2c). In the remaining animals (*n =* 2), there was no increase in HEX activity in injected SN relative to non-injected SN (data not shown). Potentially, this could be due to mistargeting of the initial viral injection, or insufficient targeting of the dissection, which could mask effects of viral HEX expression due to high baseline HEX activity in the surrounding tissue. The enzymatic upregulation was dependent on concurrent expression of both subunits, as single injections with either AAV6-HEX A or B did not produce any discernible effects on enzymatic activity (SFIG. 2A, B).

HEX acts on ganglioside GM2 by hydrolysis, generating substrate GM3 [40]. In aSYN+HEX AAV6-injected SN, there was a significant accumulation of GM3 content compared to AAV6-CTRL+aSYN-injected SN (Fig. 2d). HEX is a lysosomal enzyme, and its localization to lysosomes was verified with HEX A and LAMP-1 co-immunofluorescent labeling (SFIG. 3). Additionally, HEX overexpression was determined by quantification of # HEX A^+^ puncta/TH^+^ cell in the injected SNpc relative to the non-injected control hemisphere, which revealed a significant ~ 20-fold increase in HEX A content per TH^+^ cell (Fig. 2f). AAV6-driven expression of aSYN was robust throughout both the striatum and SNpc at 12 weeks post-injection (Fig. 2e). To rule out potential interference of the HEXA- and B viral vectors on AAV6-mediated aSYN overexpression, prior to stereological analysis and densitometry of striatal TH, successful delivery of viral transgenes was determined in all animals using experimenter-blinded visualization of human aSYN. This ensured only animals with robust transduction of human aSYN-expressing vector in the SN and STR were used, which included HEX+aSYN injected animals (SFIG. 4).

### Boosting ß-hexosaminidase activity protects the nigrostriatal dopaminergic circuit from alpha-synuclein-induced neurotoxicity

Having verified the viral-mediated upregulation of HEX enzymatic activity and concurrent accelerated GM2/GM3 conversion, the impact of this enzymatic boost on AAV6-CTRL+aSYN associated loss of nigrostriatal dopaminergic content was assessed by histological labeling of the enzyme TH in the rat SNpc. Nigrostriatal dopaminergic projections terminate in the dorsolateral aspect of the striatal caudate-putamen [4], and the integrity of these synapses are integral for proper functioning of dopamine signaling in motor control.

**Fig. 3:**
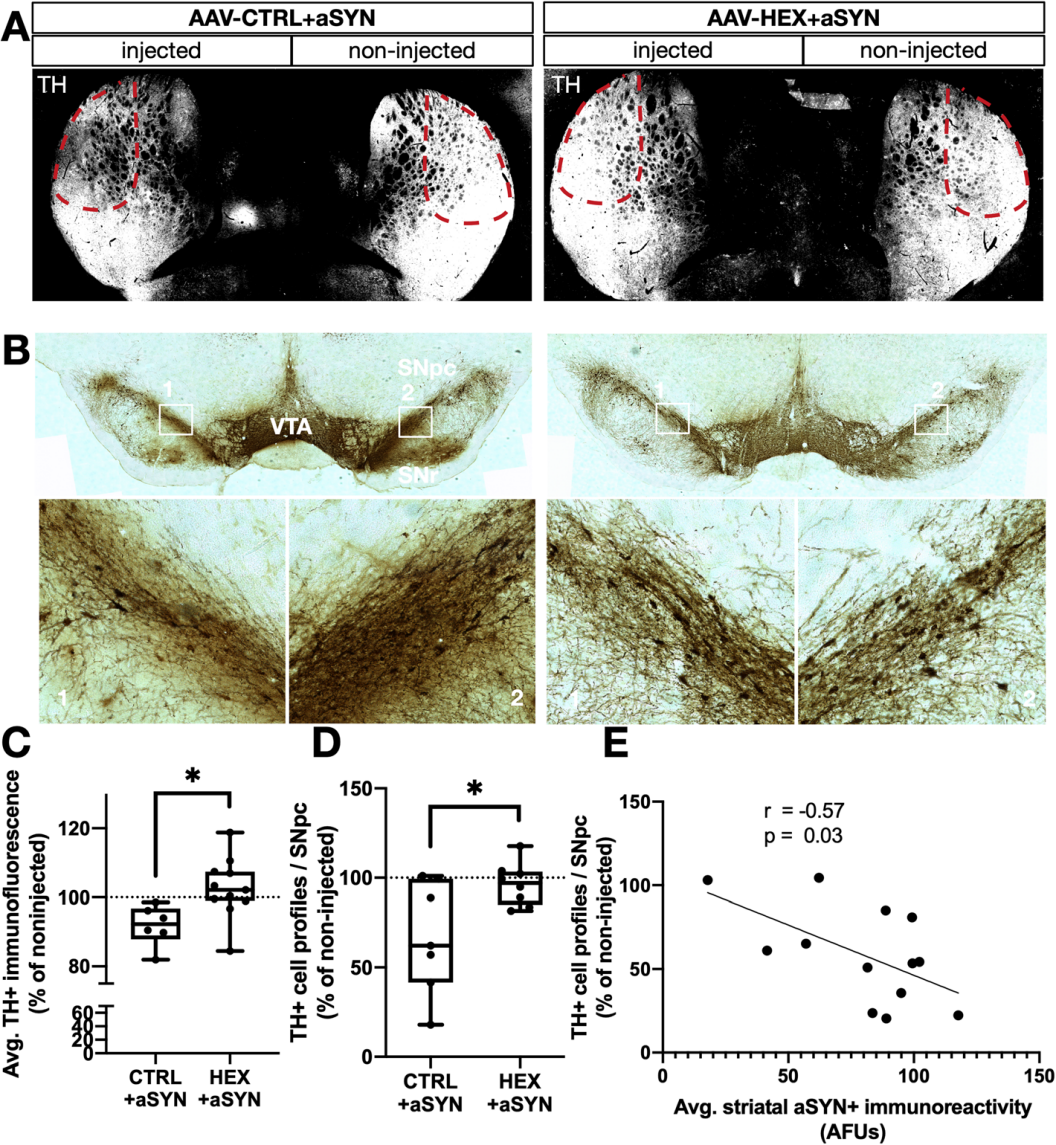
HEX co-expression rescues aSYN-associated loss of nigrostriatal TH content. **a** Representative immunofluorescent micrograph showing TH (greyscale) in the striata of rats injected with AAV6-CTRL+aSYN or aSYN+HEX. Dashed red lines indicate the dorsolateral aspect of the caudate-putamen (**b**) Representative micrograph of TH-DAB stained SNpc in rats injected with AAV6-CTRL+aSYN or aSYN+HEX. Boxed inserts are shown in higher magnification below. (**c**) Densitometric quantifications of average TH+ immunofluorescence intensity measured in the indicated regions, presented as in (**a**). *:*p* < 0.05, Welch’s t-test, *n* = 6 and 9 / group). **d** Unbiased stereological estimates of # TH+ cell profiles per SNpc, expressed as % of the non-injected hemisphere per animal (dotted line). *:*p <* 0.05, Welch’s t-test, *n =* 6 and 9 / group). Data represent median, 95%CI and range, with individual data points shown. **e** Pearson’s correlation analysis of TH+ cell counts from (**d**) with average striatal aSYN^+^ immunofluorescence (AFUs) in the region indicated in (**a**) (*N* = 13)

Upon quantification, a significant ~ 10% loss of striatal TH^+^ immunoreactivity was observed in AAV6-aSYN injected dorsolateral aspects of the striatum that was rescued by co-expression with AAV6-HEX (Fig. 3a, c). Changes in TH^+^ cell bodies of the substantia nigra pars compacta SNpc was assessed by an optical fractionator stereological probe [18]. A significant loss of ~ 35% TH^+^ neurons was found in AAV6-aSYN transduced animals that was rescued by HEX co-expression (Fig. 3b, d). Lastly, when comparing cell loss in the SNpc with accumulation of overexpressed aSYN in the dorsolateral striatum as measured by average fluorescence intensity, a significant inverse correlation was identified (Fig. 3e).

### Upregulation of ß-hexosaminidase activity prevents nigrostriatal accumulation of AAV6-overexpressed alpha-synuclein

**Fig. 4:**
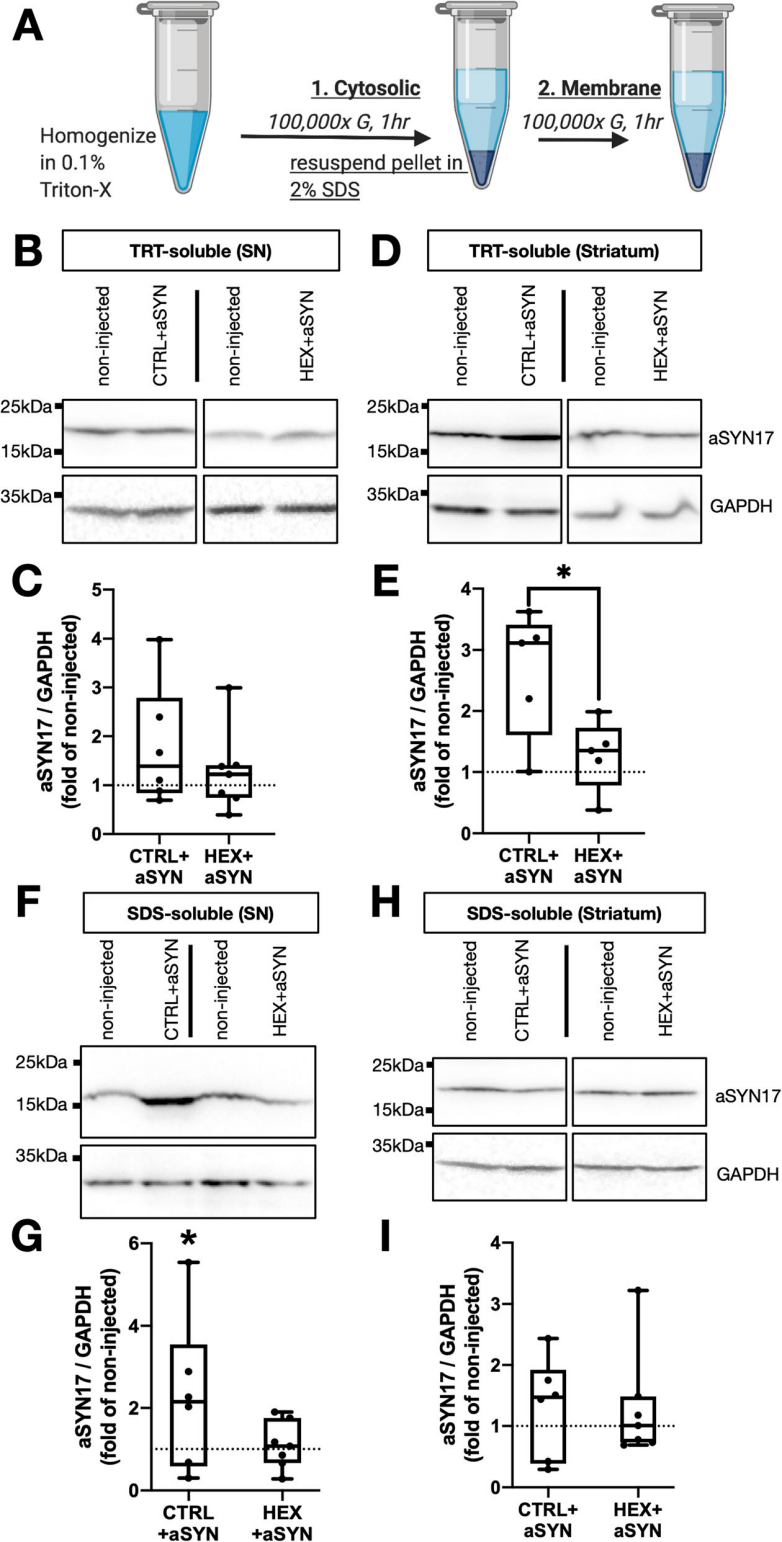
Upregulation of ß-hexosaminidase activity prevents nigrostriatal accumulation of AAV6-overexpressed alpha-synuclein. **a** Triton X-soluble (TRT) cytosolic fractions, and Triton X-insoluble/SDS-soluble membrane enriched fractions (SDS) were prepared by sequential ultracentrifugation of homogenized spot-dissected SN lysates. **b**, **d** Representative immunoblots of TRT-soluble aSYN in CTRL+aSYN and HEX+aSYN AAV6-injected SN (**b**) and striatum (**d**) relative to their non-injected control hemispheres. **c**, **e** Densitometric quantifications of aSYN normalized by GAPDH, expressed as fold change from the non-injected hemisphere per animal (dotted line) in the SN (**c**) and striatum (**e**) (*:*p* < 0.05, two-tailed t-test). **f**, **h** Representative immunoblots of SDS-soluble aSYN in CTRL+aSYN and HEX+aSYN AAV6-injected SN (**f**) and striatum (**h**) relative to their non-injected control hemispheres. **g** Densitometric quantifications of SN SDS-soluble aSYN normalized by GAPDH, compared to the non-injected control hemisphere per animal (*:*p <* 0.05, paired ratio matched sample t-test). **i** Densitometric quantifications of STR SDS-soluble aSYN normalized by GAPDH, expressed as fold of non-injected control hemisphere. *n* = 5–7 / group for all graphs

Subsequent experiments tested how the HEX enzymatic function might modulate aSYN content in these regions. Utilizing differential ultracentrifugation, spot-dissected rat ventral midbrain containing SN, and striatum from CTRL+aSYN- and aSYN+HEX injected animals were homogenized and fractionated into Triton X-soluble cytosolic fractions, and Triton X-insoluble/SDS-soluble membrane enriched fractions (Fig. 4a). Cytosol-associated aSYN was unchanged in both CTRL+aSYN and HEX+aSYN AAV6-injected SN relative to the non-injected hemispheres (Fig. 4b, c). At the level of the striatum, a significant, near 3-fold accumulation of cytosolic aSYN17 monomer could be detected, which was restored to baseline levels relative to the non-injected hemisphere in HEX+aSYN co-injected SN (Fig. 4d, e). In SDS-soluble, membrane enriched fractions, monomeric aSYN17 was significantly increased (~ 2-fold) in CTRL+aSYN injected SN relative to the non-injected hemispheres with HEX co-expression restoring levels to baseline (Fig. 4f, g). Striatal membrane-associated aSYN content was not changed in these animals (Fig. 4h, i). aSYN+ inclusions in both CTRL+aSYN and HEX+aSYN AAV6-injected SNpc were consistently co-labeled by ubiquitin (SFIG. 1B).

### ß-hexosaminidase expression decreases aSYN-association to lipid compartments

**Fig. 5:**
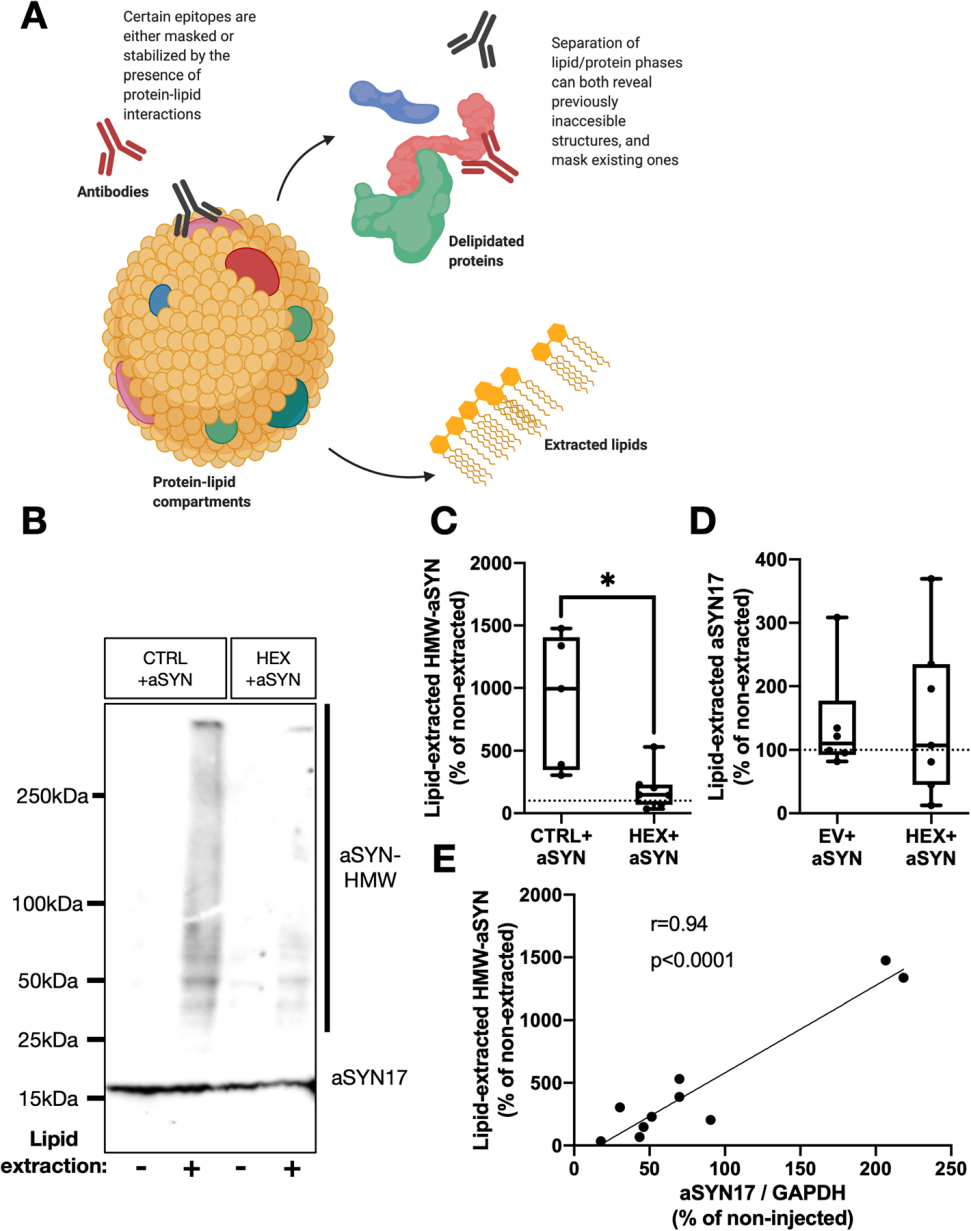
HEX co-expression prevents aSYN association with lipid compartments. **a** Illustration of the lipid extraction paradigm. Protein-lipid interactions may mask or modulate epitopes and alter detection by antibodies. Delipidation would extract these complexes and allow detection by conventional methods. **b** Representative immunoblot of aSYN17 monomer and HMW-aSYN in CTRL+aSYN or HEX+aSYN AAV6-injected rat SN, either untreated (−) or lipid extracted by heating at 65C for 18 h (+). **c** Densitometric quantifications of HMW-aSYN immunoblots from lipid extracted fractions (+) expressed as % of the lipid containing (−) fraction (*:*p <* 0.05, Welch’s t-test, *n =* 5 and 7 / group). **d** Quantifications as in (**c**) for aSYN17 monomer (*n =* 6 and 7 / group). Data represents median, 95% CI and total range with individual data points shown for all graphs. **e** Pearson’s correlation analysis of aSYN-HMW measurements as in (**c**) with SDS-soluble aSYN17 / GAPDH values from Fig. 4g (*N =* 10)

To measure association of lipids with aSYN in the injected SN of AAV6-CTRL+aSYN and AAV6-HEX+aSYN transduced rats, lipids were extracted from SDS-soluble membrane enriched (+) fractions, and aSYN immunoreactivity was compared to that of an equal amount of untreated, lipid-containing (−) homogenate in AAV6-injected dissected SN. Heating of lysates at 65C for 16 h separates protein- and lipid phases, and any protein signal that is enriched post-delipidation implies embedding of epitopes in lipid compartments [20, 45] (Fig. 5a).

Strikingly, upon lipid extraction of AAV6-CTRL+aSYN injected membrane-enriched SN homogenates, a 10-fold increase in HMW-aSYN immunoreactivity was detected compared to the untreated fractions, that was prevented by co-expression of HEX (Fig. 5b, c). Monomeric aSYN17 abundance was not altered by delipidation (Fig. 5d). When comparing lipid-extracted HMW-aSYN abundances with that of aSYN17 detectable in SDS-soluble membrane-enriched fractions within the same animals (see Fig. 4f, g), the two compartments were linearly correlated (Fig. 5e).

Interestingly, though ~ 60% of CTRL+aSYN-injected animals had robust aSYN phosphorylation in injected SNpc that was prevented by HEX co-expression (SFIG. 5A), lipid extraction did not alter the abundance of this signal (SFIG. 5B). In SD mouse whole-brain, phosphorylated aSYN (p-aSYN) was clearly detectable with no signal in age-matched WT mice (SFIG. 5C).

## Discussion

### HEX activity prevents aSYN embedding in lipid compartments

This investigation revealed that AAV6-overexpressed aSYN in the rat SNpc embedded within lipid compartments and accumulated there at a 5-fold amount compared to the accumulation of aSYN observed in both cytosolic- and membrane-enriched fractions. Boosting physiological HEX enzymatic function by AAV6-driven expression of HEX subunits A and B increased GM2/GM3 conversion and prevented the protein-lipid interactions by aSYN. Physiological aSYN strongly binds gangliosides [11, 25, 47], but previous studies in aSYN-ganglioside protein-lipid interactions have arrived at opposing conclusions regarding the outcomes of this interaction. A previous in vitro study reported binding by all a-series gangliosides - including GM1, GM2, and GM3 – to inhibit aSYN fibril formation [27], while a more recent in vitro report on the contrary found GM1 and GM3 to be potent accelerators of aSYN aggregation [13]. However, given that the primary physiological interaction of aSYN with gangliosides is localization of aSYN to synaptic terminals through binding of ganglioside-enriched lipid raft domains [12] in vitro systems are limited in providing accurate physiological conditions. Interestingly, the present report reveals a significant in vivo association of AAV6-expressed aSYN with total lipids in rat dopaminergic neurons that can be prevented by increased HEX activity; nonetheless, the lipid extraction assay does not address the specific compartments in which this interaction takes place. However, based on prior studies showing relative enrichment of gangliosides in specialized synaptic lipid raft domains, and the increased GM2/GM3 ratio reported in this study after AAV6-HEX expression, synaptic and vesicular domains are likely sites of overexpressed aSYN protein-lipid interactions. It is also noteworthy that lipid extraction produced no changes in p-aSYN detection in AAV6-CTRL+aSYN or HEX+aSYN co-injected SNpc. This is in line with prior work which demonstrated reduced lipid membrane binding by p-aSYN oligomers in vitro [31]*.*

### The effect of HEX loss of function on aSYN in Sandhoff disease models

The SD mouse line, wherein targeted disruption of the *HEXB* locus causes complete loss of HEX function and severe early-onset neuropathology [42], is an ideal system for investigating changes in physiological aSYN stemming from ganglioside GM2 accumulation. Prior studies have provided histological evidence for aSYN accumulation in the SD mouse line [9, 49], but there have been no corroborations at a biochemical level to date. In the present study, HMW-aSYN species in SD brain were 2.5-fold enriched compared to age-matched WT littermates. The current data demonstrate that loss of the ability to catabolize GM2 in the lysosome coincides with widespread HMW-aSYN formation in the brain.

In addition to the HMW-aSYN accumulation, there was a significant increase of polyubiquitin in the SD mouse brain. Accumulation of polyubiquitinated proteins can be indicative of impaired proteolysis through both the ubiquitin-proteasome- and lysosome-systems, and reduced activity of the ubiquitin C-terminal hydrolase UCH-L1 has been noted in the brains of the SD mouse model, as well as in cultured cells from numerous other lysosomal storage disorders patient samples [3]. A study by Cachón-González et al. [9] demonstrated reversibility of ubiquitin accumulation in the SD mouse with AAV6-mediated HEX overexpression, indicating that ganglioside homeostasis influencing lipid rafts may impact essential downstream cellular functions, including general ubiquitin-proteasome (UPS) and autophagy-lysosome (ALP) function, in addition to modulating aSYN protein-lipid interactions. In this current study, we did not detect changes in any UPS- or ALP-associated proteins in AAV6-CTRL+aSYN or HEX+aSYN-injected rat SNpc (data not shown). In this report, we demonstrate numerous ubiquitin+ inclusions in SD mouse SNpc that were not co-labeled by anti-aSYN antibodies, while conversely in AAV6-CTRL+aSYN and HEX+aSYN co-injected rat SNpc, the majority of aSYN+ inclusions were ubiquitin co-positive. From this, it would appear that in SD mice with complete loss of HEX function there exists generalized impairments to UPS/ALP function that is not directly associated with aSYN pathology.

### Elevated HEX activity protected the nigrostriatal dopaminergic circuit from aSYN-associated neurotoxicity

The neuroprotective potential of AAV6-HEX expression in dopaminergic neurons of the rat SNpc was determined in an AAV6-aSYN based model of synucleinopathy. Using this paradigm, upregulated HEX activity rescued dopaminergic neurons from degeneration caused by AAV6-overexpressed aSYN at the level of both SNpc cell bodies, and their terminals in the dorsolateral striatum. This synucleinopathy model is informative as it overexpresses human aSYN in the SNpc without insertion of transgenes into the animal. However, given that this is a surgically placed vector, care must be taken in addressing variability caused by the actual location and expression of the transgene. The AAV6-aSYN titers selected for infusion into the SNpc in this study produced a mild ~ 35% loss of TH^+^ neurons in the SNpc. There was an average ~ 10% reduction in striatal TH density, possibly relating to the known relative increase in striatal TH prior to degeneration in models of aSYN overexpression [22, 33]. Given this less toxic dose, there is a greater chance of measuring specific interactions between aSYN and HEX-dependent ganglioside metabolism, as neuronal loss is limited. Furthermore, there was no aSYN+ immunoreactivity detectable in SDS-insoluble SNpc homogenate fractions from these animals, showing that in this model there was no formation of insoluble fibrillar or amyloid aSYN species contributing to the pathology. This was also verified in SDS-insoluble whole-brain homogenate fractions from SD mice, which similarly had no detectable aSYN+ immunoreactivity (data not shown).

AAV6-HEX overexpression increased GM2/GM3 turnover. One interpretation is that, given the preferential enrichment of gangliosides in lipid raft domains of the plasma membrane, the observed reduced lipid binding of aSYN by HEX overexpression was mediated by increased turnover of GM2/GM3 in these compartments. However, it is probable that the impact of altered ganglioside abundance depends on the context, which in our experimental system was one of increased aSYN protein burden. In another context, in *Galgt1*^−/−^ transgenic mice lacking GM2 synthase, GM2 content is fully depleted, but - similarly to SD mice presenting with an overabundance of GM2 - they develop early HMW-aSYN formation and dopaminergic neuron degeneration, in addition to a severe motor phenotype [53]. In the *Galgt1*^−/−^ mice, administration of a brain penetrant GM1 analog rescued these deficits, which demonstrates the crucial importance of a neuronal ganglioside on physiological aSYN function and overall cellular viability. In human PD SNpc, levels of major ganglioside biosynthesis enzymes are decreased in neuromelanized dopaminergic neurons, but not proximal, non-melanized cells [43], indicative of cell-type specific impairments in ganglioside metabolism. These findings were confirmed in a separate study, which by HPLC measurements of human post-mortem PD-patient SN found all major gangliosides to be significantly reduced relative to age-matched healthy subject controls, which in PD subjects were reduced in an age-dependent fashion [17]. Cerebrospinal fluid measurements taken from these same patients revealed significantly increased GM3/GM2 ratios, which could indicate a compensatory drive towards enrichment of these gangliosides through alternate metabolic harvesting pathways, as overall HEX activity in the SN of PD patients was decreased. The critical importance of GM3 homeostasis on neuronal development and function can be gleaned from studies in *Sat-1*^−/−^ GM3 synthase deficient mice with complete GM3 depletion, which present with developmental neurodegenerative abnormalities and numerous behavioral deficits [30, 55].

In summary, this report characterizes the neuroprotective effect of increased HEX enzymatic activity on aSYN-induced degeneration of dopaminergic neurons in vivo*,* which appears to be mediated - at least in part - by reduced aSYN in lipid compartments. Future work should aim to identify the specific lipid compartments in which GM2/GM3 content appear to modulate aSYN incorporation, to better understand the cellular processes most vulnerable to impaired ganglioside homeostasis in disorders of the central nervous system.

## Supplementary information

**Additional file 1: Supplementary Fig. 1.** aSYN-ubiquitination in the SD-TG mouse model and in AAV-aSYN-injected rat SNpc. (**A**) Representative micrograph of aSYN (green) and ubiquitin (magenta) in age-matched WT- and SD-TG mouse SNpc. Filled arrowheads indicate ubiquitin+ aSYN+ inclusions. Open arrowheads indicate ubiquitin+ aSYN- inclusions. Boxed inserts are shown in higher magnification with orthogonal view on right. Scale bar = 15um. **(B)** Representative micrograph of aSYN (green) and ubiquitin (magenta) in non-injected and AAV-CTRL+aSYN and HEX+aSYN injected rat SNpc. Arrowheads indicate ubiquitin+ aSYN+ inclusions. Boxed inserts are shown in higher magnification with orthogonal view on right. Scale bar = 15um. **Supplementary Fig. 2.** AAV6-mediated expression of HEX subunits A and B alone does not elicit upregulation of enzymatic activity (**A**) HEX activity histochemical detection assay (red precipitate) in a rat injected with AAV6 expressing HEX subunit B. (SNr = substantia nigra pars reticulata, SNpc = substantia nigra pars compacta, VTA = ventral tegmental area). Boxed inserts are shown in higher magnification below. **(B)** High magnification inserts of tissue prepared as in **(A)**, in SNpc injected (1) with AAV6-HEX A, and the non-injected control hemisphere (2). **Supplementary Fig. 3.** Overexpressed HEX localizes to lysosomes in transduced TH^+^ neurons of the rat SNpc. Representative immunofluorescent micrograph showing HEX subunit A (red) and LAMP-1 (greyscale) in TH+ dopaminergic neurons (green) in the SNpc of AAV6-HEX injected- and non-injected hemispheres. Dotted outline represents TH+ cell profile. Boxed insert is shown in orthogonal view (YZ). **Supplementary Fig. 4.** Representative immunofluorescent micrograph showing AAV6-overexpressed human WT aSYN (magenta) in dopaminergic neurons (TH+) (green) in the rat striatum and SNpc of an animal injected with AAV6-aSYN, HEXA and HEXB (STR = striatum, VTA = ventral tegmental area, SNpc = substantia nigra pars compacta, SNr = substantia nigra pars reticulata.) **Supplementary Fig. 5.** Phosphorylated aSYN is a feature of both AAV-mediated aSYN overexpression and HEX loss-of-function. (**A**) Immunoblot of S129-phosphorylated aSYN (p-aSYN) in CTRL+aSYN and HEX+aSYN AAV-injected and non-injected rat SNpc. GAPDH is shown as loading control. **(B)** Immunoblot of p-aSYN CTRL+aSYN-injected SNpc containing lipids (+) or after lipid extraction at 65C for 16 h(−). **(C)** Immunoblot of p-aSYN in age-matched WT- or Sandhoff transgenic (SD) mouse whole-brain. GAPDH is shown as loading control.

## Data Availability

The datasets generated during and/or analyzed during the current study are available from the corresponding authors. The datasets are stored as electronic records.

## Abbreviations

AAV: Adeno-associated virus
aSYN: α-synuclein
GCase: Glucocerebrosidase
GD: Gaucher disease
HEX: β-hexosaminidase
HMW: High molecular weight
LSDs: Lysosomal storage disorders
SD: Sandhoff disease
PD: Parkinson’s disease
SNpc: Substantia nigra pars compacta
