# Supplementary material for: Upregulating β-hexosaminidase activity in rodents prevents α-synuclein lipid associations and protects dopaminergic neurons from α-synuclein-mediated neurotoxicity
Source: Acta Neuropathol Commun. 2020 Aug 6;8:127. doi: 10.1186/s40478-020-01004-6 (PMC7409708; doi:10.1186/s40478-020-01004-6)
Supplement: Supplementary file 1 — Additional file 1: Supplementary Fig. 1. aSYN-ubiquitination in the SD-TG mouse model and in AAV-aSYN-injected rat SNpc. (A) Representative micrograph of aSYN (green) and ubiquitin (magenta) in age-matched WT- and SD-TG mouse SNpc. Filled arrowheads indicate ubiquitin+ aSYN+ inclusions. Open arrowheads indicate ubiquitin+ aSYN- inclusions. Boxed inserts are shown in higher magnification with orthogonal view on right. Scale bar = 15um. (B) Representative micrograph of aSYN (green) and ubiquitin (magenta) in non-injected and AAV-CTRL+aSYN and HEX+aSYN injected rat SNpc. Arrowheads indicate ubiquitin+ aSYN+ inclusions. Boxed inserts are shown in higher magnification with orthogonal view on right. Scale bar = 15um. Supplementary Fig. 2. AAV6-mediated expression of HEX subunits A and B alone does not elicit upregulation of enzymatic activity (A) HEX activity histochemical detection assay (red precipitate) in a rat injected with AAV6 expressing HEX subunit B. (SNr = substantia nigra pars reticulata, SNpc = substantia nigra pars compacta, VTA = ventral tegmental area). Boxed inserts are shown in higher magnification below. (B) High magnification inserts of tissue prepared as in (A), in SNpc injected (1) with AAV6-HEX A, and the non-injected control hemisphere (2). Supplementary Fig. 3. Overexpressed HEX localizes to lysosomes in transduced TH+ neurons of the rat SNpc. Representative immunofluorescent micrograph showing HEX subunit A (red) and LAMP-1 (greyscale) in TH+ dopaminergic neurons (green) in the SNpc of AAV6-HEX injected- and non-injected hemispheres. Dotted outline represents TH+ cell profile. Boxed insert is shown in orthogonal view (YZ). Supplementary Fig. 4. Representative immunofluorescent micrograph showing AAV6-overexpressed human WT aSYN (magenta) in dopaminergic neurons (TH+) (green) in the rat striatum and SNpc of an animal injected with AAV6-aSYN, HEXA and HEXB (STR = striatum, VTA = ventral tegmental area, SNpc = substantia nigra pars compacta, SNr = su [file 40478_2020_1004_MOESM1_ESM.docx]

**Upregulating β-hexosaminidase activity in rodents prevents α-synuclein lipid associations and protects dopaminergic neurons from α-synuclein-mediated neurotoxicity.**

by Oeystein R. Brekk^1^, Joanna A. Korecka^1,#^, Cecile C. Crapart^1^, Mylene Huebecker^2,##^, Zachary K. MacBain^1^, Sara Ann Rosenthal^1^, Miguel Sena-Esteves^3^, David A. Priestman^2^, Frances M. Platt^2^, Ole Isacson^1,*^ & Penelope J. Hallett^1,*^

1. Neuroregeneration Institute, McLean Hospital / Harvard Medical School, Belmont MA, USA.

2. Department of Pharmacology, University of Oxford, Oxford, UK.

3. Department of Neurology, University of Massachusetts Medical School, Worcester MA, USA.

#. Current address: Department of Neurology, Brigham and Women’s Hospital, Boston MA, USA.

##. Current address: Institute of Innate Immunity, Medical Faculty, University of Bonn, Bonn, Germany.

*. Corresponding authors:

Ole Isacson (isacson@hms.harvard.edu),

Penelope J. Hallett (phallett@mclean.harvard.edu)

**Supplementary information**

**Supplementary Figure 1. aSYN-ubiquitination in the SD-TG mouse model and in AAV-aSYN-injected rat SNpc. (A)** Representative micrograph of aSYN (green) and ubiquitin (magenta) in age-matched WT- and Sandhoff disease (SD)-TG mouse SNpc. Filled arrowheads indicate ubiquitin^+^aSYN^+^ inclusions. Open arrowheads indicate ubiquitin^+^aSYN^-^ inclusions. Boxed inserts are shown in higher magnification with orthogonal view on right. **(B)** Representative micrograph of aSYN (green) and ubiquitin (magenta) in non-injected and AAV-CTRL+aSYN and HEX+aSYN injected rat SNpc. Arrowheads indicate ubiquitin^+^aSYN^+^ inclusions. Boxed inserts are shown in higher magnification with orthogonal view on right. Scale bars = 15µm.


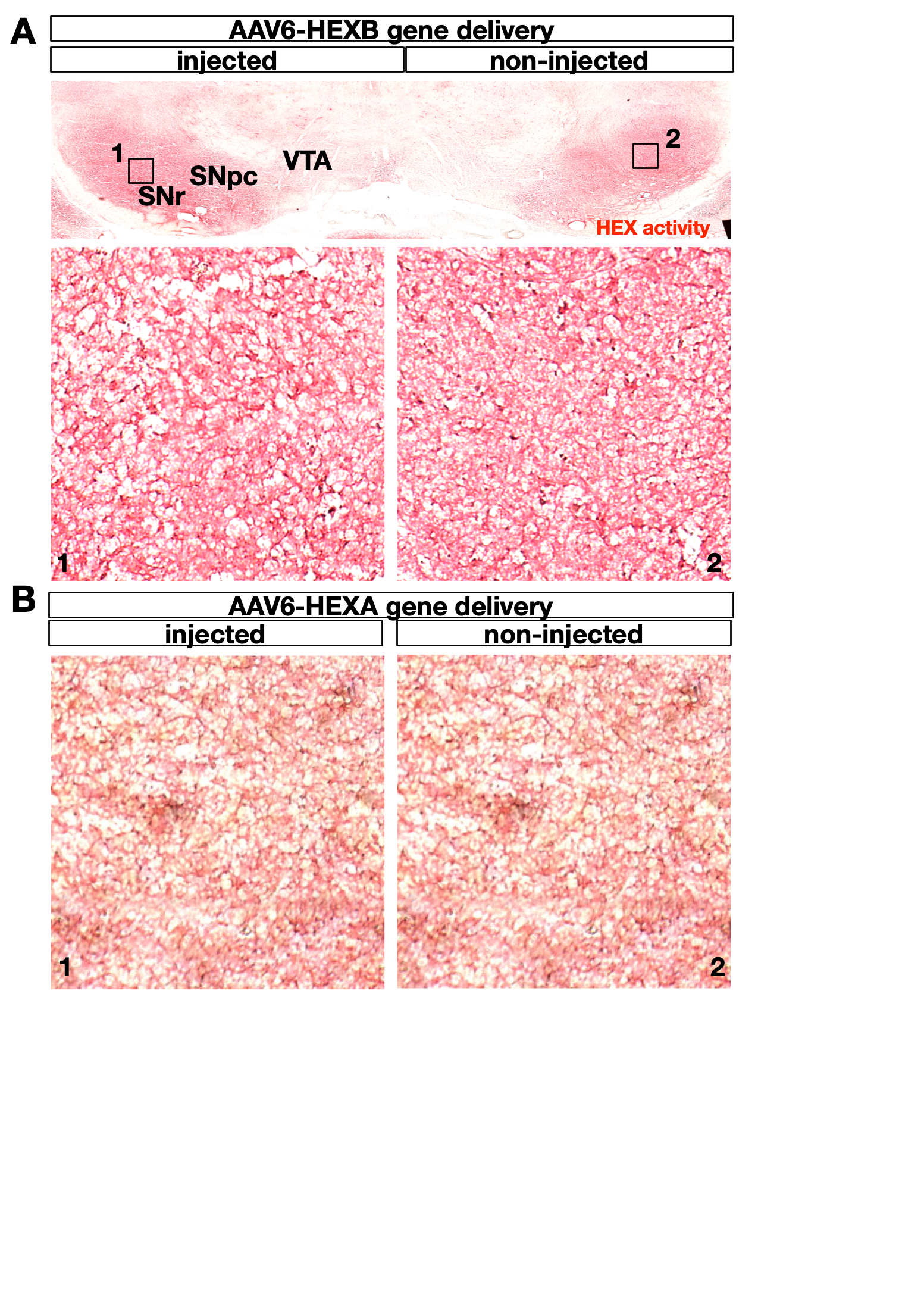


**Supplementary Figure 2. AAV6-mediated expression of HEX subunits A and B alone does not elicit upregulation of enzymatic activity (A)** HEX activity histochemical detection assay (red precipitate) in a rat injected with AAV6 expressing HEX subunit B. (SNr = substantia nigra pars reticulata, SNpc = substantia nigra pars compacta, VTA = ventral tegmental area). Boxed inserts are shown in higher magnification below. **(B)** High magnification inserts of tissue prepared as in **(A)**, in SNpc injected (1) with AAV6-HEX A, and (2) the non-injected control hemisphere.

**Supplementary Figure 3. Overexpressed HEX localizes to lysosomes in transduced TH^+^ neurons of the rat SNpc.**  Representative immunofluorescent micrograph showing HEX subunit A (red) and LAMP-1 (greyscale) in TH^+^ dopaminergic neurons (green) in the SNpc of AAV6-HEX injected- and non-injected hemispheres. Dotted outline represents TH^+^ cell profile. Boxed insert is shown in orthogonal view (YZ).


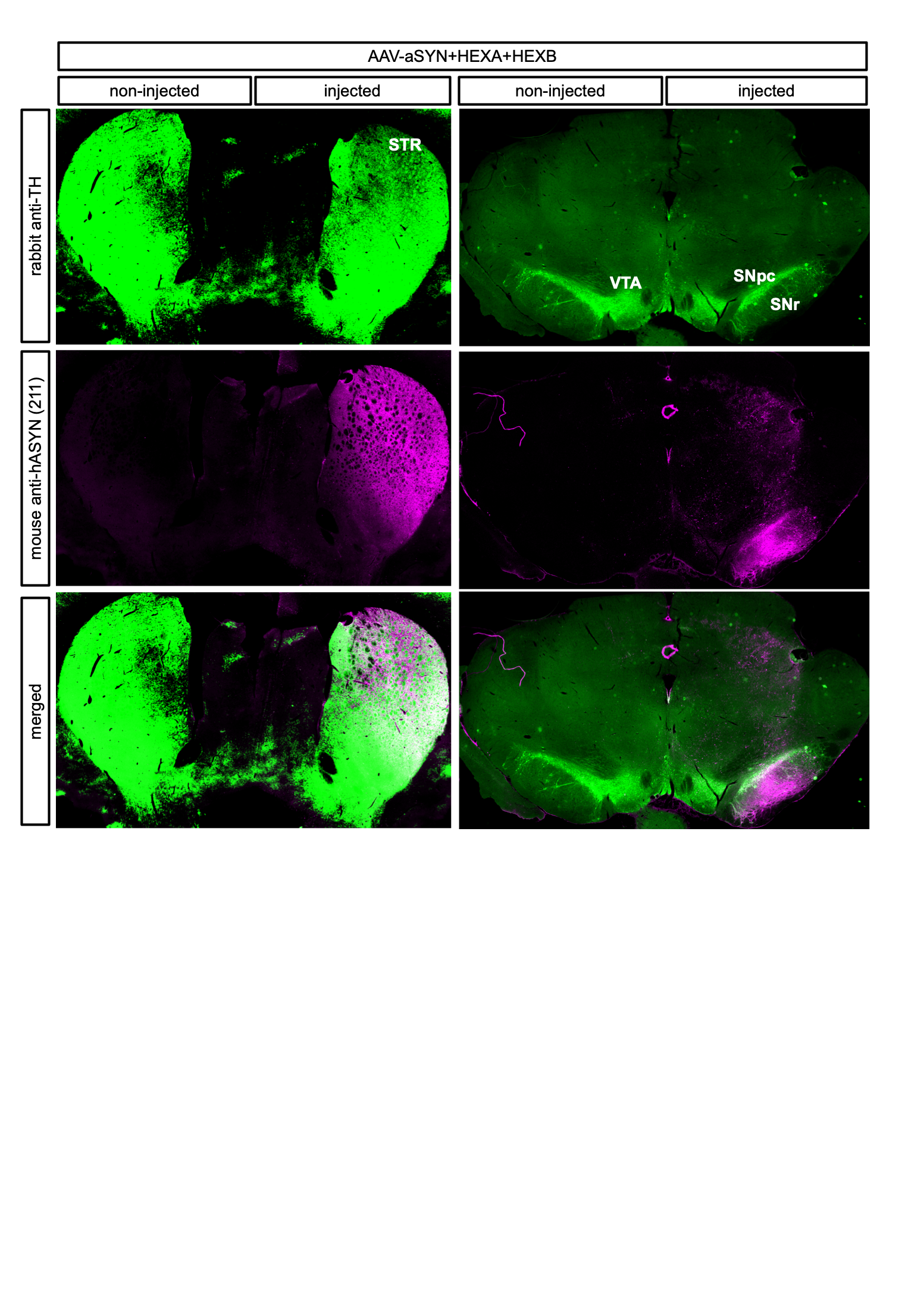


**Supplementary Figure 4.** Representative immunofluorescent micrograph showing AAV6-overexpressed human WT aSYN (magenta) in dopaminergic neurons (TH+) (green) in the rat striatum and SNpc of an animal injected with AAV6-aSYN, HEXA and HEXB (STR = striatum, VTA = ventral tegmental area, SNpc = substantia nigra pars compacta, SNr = substantia nigra pars reticulata.)


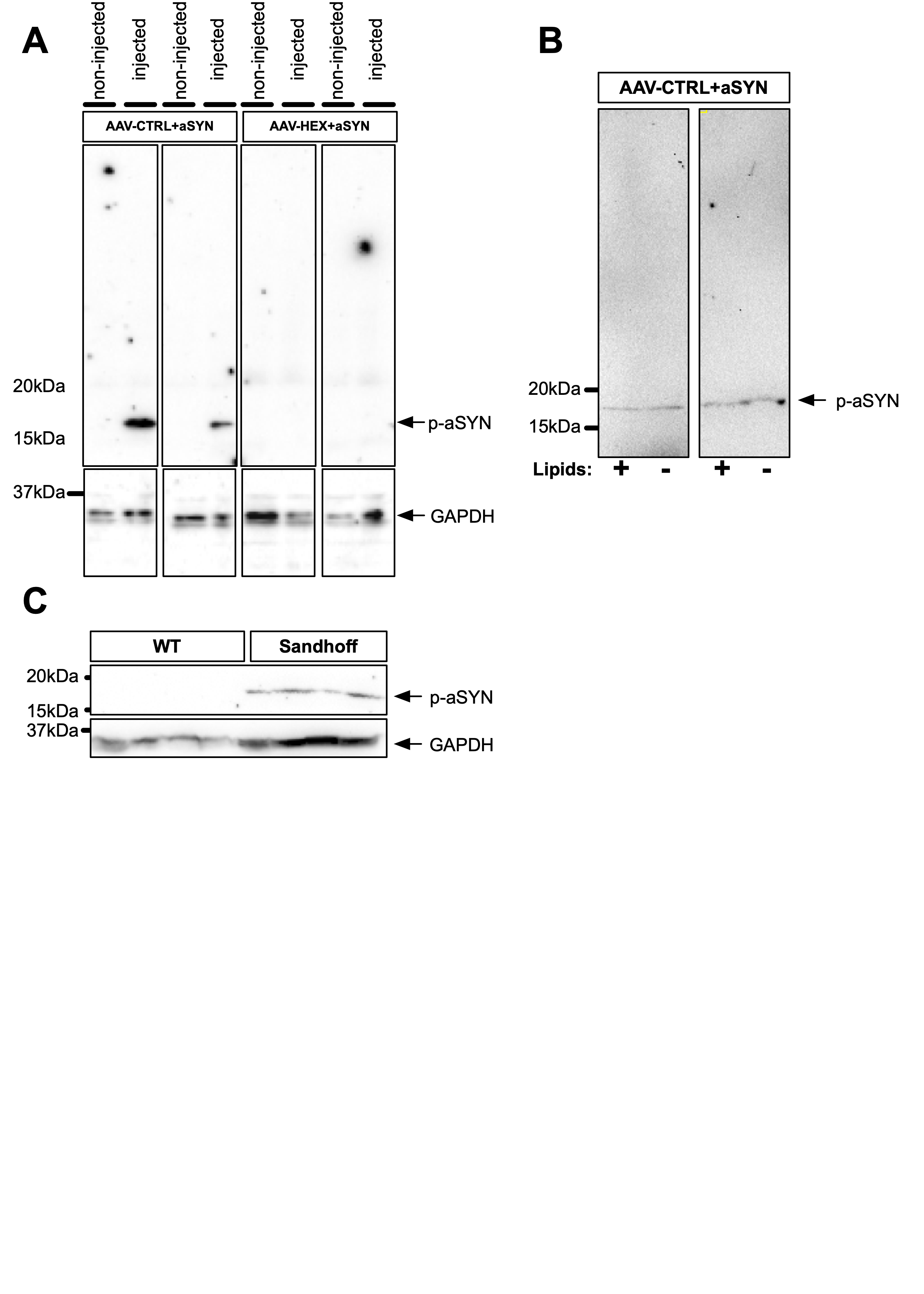


**Supplementary Figure 5. Phosphorylated aSYN is a feature of both AAV-mediated aSYN overexpression and HEX loss-of-function. (A)** Immunoblot of S129-phosphorylated aSYN (p-aSYN) in CTRL+aSYN and HEX+aSYN AAV-injected and non-injected rat SNpc. GAPDH is shown as loading control. **(B)** Immunoblot of p-aSYN CTRL+aSYN-injected SNpc containing lipids (+) or after lipid extraction at 65C for 16 hours (-). **(C)** Immunoblot of p-aSYN in age-matched WT- or Sandhoff transgenic (SD) mouse whole-brain. GAPDH is shown as loading control.
